# High ALG1 Expression Is Correlated With Poor Prognosis and the Immune Microenvironment in Glioma

**DOI:** 10.1111/jcmm.71142

**Published:** 2026-04-19

**Authors:** Shuxiang Li, Yang Xia, Qiang Liu, Xin Qian, Anhong Liu, Jia Wang, Xinyu Wang, Di Zuo, Jing Zhang, Jinhai Ma, Peng Gao

**Affiliations:** ^1^ Department of Paediatrics General Hospital of Ningxia Medical University Yinchuan Ningxia Province China; ^2^ Ningxia Key Laboratory of Cerebrocranial Diseases Ningxia Medical University Yinchuan Ningxia Province China; ^3^ Department of Neurosurgery General Hospital of Ningxia Medical University Yinchuan Ningxia Province China; ^4^ The First People’s Hospital of Shizuishan Shizuishan Ningxia Province China; ^5^ Department of Nerve Electrophysiology General Hospital of Ningxia Medical University Yinchuan Ningxia Province China; ^6^ Institute of Medical Sciences, Reproductive Medicine Center General Hospital of Ningxia Medical University Yinchuan Ningxia Province China

**Keywords:** ALG1, glioma, immune, prognosis, tumour microenvironment

## Abstract

Glioma is the most prevalent primary tumour in the central nervous system. This study investigates ALG1 expression in glioma and its clinical significance. Using the TCGA and CGGA databases, a bioinformatics analysis examines ALG1 expression, its prognostic value, and its link to the immune microenvironment. Clinical samples are analysed using RT‐qPCR, Western blotting, and immunohistochemistry/fluorescence for expression validation. Single‐cell RNA sequencing (scRNA‐seq) assesses ALG1's role in cell state transitions. In vitro, wound healing assays are performed after ALG1 knockdown with siRNA, and in vivo analysis uses an in situ mouse model. The pan‐cancer analysis revealed that ALG1 is significantly overexpressed in varioustumours, including glioblastoma (GBM), and is linked to poor patient outcomes. A prognostic nomogram based on ALG1 levels accurately predicted 1‐, 3‐, and 5‐year survival rates for glioma patients. In clinical samples, ALG1 was highly expressed at both mRNA and protein levels, correlating with tumour grade. Single‐cell analysis showed increasing ALG1 expression from non‐tumorigenic to malignant states, especially in malignant cells. ALG1 overexpression altered tumour microenvironmentsignalling, changing IL10 and CYPA pathways associated with immune suppression. Knocking down ALG1 significantly reduced glioma cell migration and downregulated EMT‐related proteins like N‐cadherin, β‐catenin, and Vimentin. This study highlights ALG1 as a key prognostic biomarker and therapeutic target for glioma, promoting malignancy through increased cell migration, EMT modulation, and an altered immunosuppressive microenvironment.

## 1 Introduction

Gliomas are the most prevalent primary malignant tumours of the central nervous system (CNS), originating from glial cells and accounting for approximately 22.9% of all tumours [1]. With the development of molecular biology, novel therapeutic strategies, including immunotherapy, targeted therapy, and tumour‐treating fields (TTFields) therapy, have been proposed [2]. However, the prognosis of glioma patients remains unsatisfactory, with glioblastoma (GBM) as an example, with a median overall survival (OS) of only 14–16 months [3, 4, 5]. The poor prognosis of patients is also attributed to the pronounced heterogeneity and the epigenetic alterations present in gliomas [6, 7]. Furthermore, the presence of the physiological blood‐brain barrier (BBB) significantly hampers the delivery and efficacy of therapies, particularly targeted drugs and immunotherapies [8, 9]. Therefore, in‐depth exploration of the molecular mechanisms of glioma is important for improving clinical diagnosis and treatment.

The 2021 fifth edition of the World Health Organization (WHO) classification incorporated molecular markers into the diagnostic criteria for gliomas to improve diagnostic precision and enable more effective stratified treatment [10]. The isocitrate dehydrogenase (IDH) mutations and 1p/19q codeletion have become key diagnostic markers of a favourable prognosis for glioma patients [10, 11]. However, gliomagenesis is a complex, multifactorial process that involves dysregulation of signalling pathways, metabolic reprogramming, and immune microenvironment [12, 13]. Therefore, the identification of additional molecular biomarkers is critical for prognostic assessment and individualised therapy.

Glycosylation, as one of the most prevalent posttranslational modifications (PTMs), has recently gained increased attention in cancer research [14]. *N*‐glycosylation plays a critical role in the regulation of key biological processes, including cell adhesion, protein stability, signal transduction, and immune escape [15], and complex biosynthetic processes are mediated by a series of glycosyltransferases [16]. Among these, asparagine‐linked glycosylation protein 1 homologue (ALG1) encodes a β‐1,4‐mannosyltransferase, where it catalyses the addition of a second mannose residue to the lipid‐linked oligosaccharide precursor during the early stages of N‐glycan synthesis [17]. Recent studies have demonstrated that ALG1 plays a critical role in various tumours [18, 19, 20]. For example, ALG1 downregulation affects glycosylation of N‐cadherin in hepatocellular carcinoma, which promotes tumour metastasis [18]. Similarly, ALG1 expression has been implicated as a potential prognostic biomarker in colorectal [19] and gastric adenocarcinomas [20]. However, the role of ALG1 in glioma has not been fully elucidated.

In this study, we investigated the role of ALG1 in glioma through pan‐cancer data mining, survival analysis, immune infiltration profiling, single‐cell trajectory detection, and in vitro and vivo functional assays. Our findings revealed that ALG1 is significantly overexpressed in glioma and is correlated with the tumour microenvironment (TME) and malignant progression. These results provide powerful evidence that ALG1 is a promising diagnostic and prognostic biomarker in glioma.

## 2 Materials and Methods

### 2.1 Data Acquisition and Pan‐Cancer ALG1 Differential Expression

Pan‐cancer analysis data of 683 patients with primary glioma, including those with GBM and low‐grade glioma (LGG), were downloaded from the TCGA database. Furthermore, we screened 615 glioma patients whose complete clinical information was available in a test database within the CGGA to analyse the expression level of ALG1 and OS. To ensure data comparability, we matched samples of tumour and adjacent normal tissues from anatomically homologous sites within the same population in the TCGA database that were systematically selected. According to the status of the samples, we used the Wilcoxon rank‐sum test for unpaired samples and the Wilcoxon signed‐rank test for paired samples. These databases are publicly accessible, and thus, approval from the local ethics committee was not required.

### 2.2 Prognostic Analysis and Prediction Model Construction

Glioma patients were stratified into high‐ and low‐expression groups according to the median ALG1 expression level, and Kaplan–Meier survival analysis was performed. Univariate Cox regression analysis was performed for the TCGA cohort to construct the optimal prognostic model, and the C‐index was calculated to evaluate the pretesting capability of the module, which was greater than 0.50, demonstrating meaningful accuracy. The TCGA cohort subsequently served as the training set, while the CGGA cohort served as the validation set. We compared the expression of ALG1 in gliomas between different clinical characteristics, such as age (< 65, ≥ 65), sex (female, male), grade (WHO 2, WHO 3, and WHO 4), IDH status (mutant, wild‐type), 1p/19q status (codeletion, non‐codeletion), and MGMT status (methylated, unmethylated). The MGMT promoter status was obtained from the clinical annotation files of the TCGA and CGGA datasets. In the initial datasets, MGMT promoter methylation was assessed through DNA pyrosequencing analysis of CpG sites within the MGMT promoter region [21]. Following the exclusion of cases lacking complete clinicopathological information, the TCGA and CGGA cohorts were incorporated into the nomogram analysis. A nomogram incorporating the above clinical variables was developed using the rms R package to estimate the 1‐, 3‐, and 5‐year OS probabilities. In addition, time‐dependent receiver operating characteristic (ROC) curve analysis was conducted to evaluate the prognostic value.

### 2.3 Tumour Sample Collection

In all six glioma tissue samples and corresponding peritumoral tissues (2 WHO grade 2, 2 WHO grade 3, and 2 GBM) were collected from patients who underwent surgical resection at the General Hospital of Ningxia Medical University, China, between January 2025 and August 2025. The clinicopathological data are summarised in Table S1. All the tissue samples were immediately snap‐frozen in liquid nitrogen after surgery. Then, we performed immediate quantitative reverse transcription PCR (RT‐qPCR) and Western blotting (WB). In addition, 52 paraffin blocks were judged adequate for histological evaluation. The clinical data of the patients are provided in Table S2. This study complied with the Declaration of Helsinki, and informed consent was obtained from all patients before the study began. The Ethics Committee of the General Hospital of Ningxia Medical University approved the study (No. KYLL‐2025‐2249).

### 2.4 Immunohistochemistry and Immunofluorescence Staining

ALG1 protein expression was determined by IHC staining in glioma. In addition, we examined the colocalisation of CD4, CD8 and PDL1 with ALG1 by immunofluorescence (IF). Tissue sections were deparaffinised, rehydrated, and subjected to antigen retrieval by heating the slides in sodium citrate buffer. Endogenous peroxidase activity was blocked by incubation with 3% hydrogen peroxide for 10 min, after which the slides were washed three times with PBS. The samples were then incubated with rabbit anti‐ALG1 (1:500, 12,548–1‐AP, Abmart, China) alone for IHC staining and anti‐ALG1 in addition to CD4 (1:500, 44,038–1, Signalway, USA), CD8 (1:500, 58,251, Signalway, USA) and PDL1 (1:500, 27,584, Signalway, USA) antibodies for IF overnight at 4°C. This was followed by a 2‐h incubation with goat anti‐rabbit IgG HRP (1:1000, B900210, Proteintech, China) and Alexa Fluor‐labelled secondary antibodies (1:1000, 150,115, Abcam, USA), respectively. For IHC staining with 3,3′‐diaminobenzidine. The nuclei in the IF‐stained slides were stained with DAPI. Images of stained slides were captured with a microscope (Leica, Germany), and the results were analysed using ImageJ.

### 2.5 RT‐qPCR Detection and Western Blot Experiment

Total RNA was extracted from cells and fresh glioma tissues using a kit (Beyotime, China). Reverse transcription was performed with 1 μg of total RNA (ThermoFisher, USA). The primers used for GAPDH and ALG1 were synthesised by Sangon Biotech. RT‐qPCR was performed in triplicate for each sample using the Quantity‐Nova SYBR Green PCR system. Relative changes in transcripts were calculated according to the 2^^(‐ΔΔCt)^ method. Tissues from six glioma patients were homogenised in lysis buffer (Servicebio, China). The primer sequences were as follows: *GAPDH* (Human, forward: CAGGAGGCATTGCTGATGAT; reverse: GAAGGCTGGGGCTCATTT) and *ALG1* (Human, forward: TGGAAGCAAGCTCGTCATTGA; reverse: TGGTAACACACAGGTTCAGGT); *GAPDH* (Mouse, forward: AGGTCGGTGTGAACGGATTTG; reverse: TGTAGACCATGTAGTTGAGGTCA) and *ALG1* (Mouse, forward: TCAGGCAGTGTACTTGCTGTGG; reverse: CCACAAACCAGCAGACGGCAAT).

The protein concentration was determined using a BCA kit (KeyGEN, China), after which equal amounts of protein were separated by 10% SDS‐PAGE. After transfer and blocking, the membranes were incubated overnight with antibodies against ALG1 (1:3000, 12,548–1‐AP, Abmart, China), β‐catenin (1:3000, 8,480, CST, USA), N‐cadherin (1:1000, A19083, Abconal, China), and vimentin (1:1000, A19607, Abconal, China). To confirm equal loading, β‐actin (1:5000, 38,063, SAB, USA) or GAPDH (1:5000, GB15002‐100, Signalway, USA) antibodies served as loading controls.

### 2.6 Cell Culture, siRNA Interference and Wound Healing Assay

GL261, Human HA, U251 and A172 cell lines were acquired from the Chinese Academy of Sciences and cultured in Dulbecco's modified Eagle's medium (DMEM; Gibco, USA) supplemented with 10% fetal bovine serum (FBS; BI, Israel) and 1% streptomycin–penicillin at 37°C in a 5% CO_2_ atmosphere. Small interfering RNA (siRNA) targeting human NC siRNA (sense: UUCUCCGAACGUGUCACGUTT; antisense: ACGUGACACGUUCGGAGAATT), ALG1‐siRNA1 (sense: GGGCGGCCCGGCAUGUAGUTT; antisense: ACUACAUGCCGGGCCGCCCTT), and ALG1‐siRNA2 (sense: GCACGGCUUCUCGGUGACCTT; antisense: GGUCACCGAGAAGCCGUGCTT) were commercially synthesised (GenePharm, China) and transfected into cells using siRNA Mate Plus transfection reagent. Approximately 10^6^ U251 and A172 cells transfected with the siRNAs described above were seeded in 6‐well plates. Vertical scratches were generated on the monolayer when the cells reached 95% confluence. Serum‐free medium was then used to maintain the cells. The wound gaps were measured at 0 h, 24 h, and 48 h by ImageJ.

### 2.7 Intracranial Model

An intracranial tumour mouse model was established to investigate the effect of ALG1 in vivo. Four‐week‐old male C57BL/6J mice (*n* = 20) were purchased from Ningxia Medical University Institutional Animal Center and raised in SPF conditions. Twenty C57 mice were divided into two experimental groups: The experimental group (*n* = 10) and the control group (*n* = 10). All surgical procedures were performed under isoflurane anaesthesia to minimise animal suffering. Then, the skull was drilled with a needle tip 3.5 mm from the cerebral midline and 2 mm frontal to the coronal suture. Five microliters of the GL261 cell suspension or PBS were injected at a depth of 3 mm from the brain surface. Four weeks post‐injection was considered the terminal point. The whole brains of the mice were removed andphotographed, then fixed in 4% paraformaldehyde, and embedded in paraffin for H&E and IHC staining. All experiments with animals were approved by the Ningxia Medical University Institutional Animal Care and Use Committee (Approval No. SCXK (Ning) 2020–0001, Quality Certificate No. 10752309202500338). Humane endpoints were applied according to institutional guidelines, and all efforts were made to reduce the number of animals used and to alleviate their pain and distress.

### 2.8 Identification of Differentially Expressed Genes and Functional Enrichment Analysis

Differentially expressed genes (DEGs) between the high and low ALG1 expression groups were identified among 615 glioma patients in the TCGA cohort, with the median ALG1 expression level used as the cutoff. DEG analysis was performed on HTSeq count data using the DESeq2 package [22]. |log2fold‐change (FC)| > 1.5 and adjusted *p* < 0.05 were considered threshold values for DEGs, which were subsequently visualised in a volcano plot. To further explore the potential mechanism of ALG1, we conducted GO and KEGG pathway analyses using the clusterProfiler package [23], and *p*‐values were adjusted according to the Benjamini‐Hochberg method. A normalised enrichment score (NES) > 1 and a false discovery rate (FDR) < 0.05 were considered significant.

### 2.9 Evaluation of the Function of ALG1 in Immune Cell Infiltration in the Glioma Microenvironment

Single‐sample gene set enrichment analysis (ssGSEA) based on the expression of 141 immune signature genes was performed to compute immune scores to predict the level of infiltrating immune cells. The abundance of infiltrating immune cells was assessed using the TIMER and EPIC algorithms [24, 25]. Pearson correlation analysis was conducted to evaluate the associations between the risk score and immune cell infiltration levels. Additionally, we calculated immune scores for all the subjects using the Expression data algorithm, which allowed us to deduce the composition of stromal and immune cells in tumour samples based on the transcriptional profiles of cancer samples [26]. Then, we performed glioma clustering according to immune‐related genes; nonnegative matrix factorisation (NMF), a commonly used dimensionality reduction technique for high‐dimensional genomic data, was applied to identify molecular subtypes. We obtained a list of immune‐related genes from the ImmPort database [27]. Immune‐related gene expression profiles were extracted from the TCGA dataset and subjected to NMF clustering. The optimal number of clusters was determined according to the cophenetic correlation coefficients.

### 2.10 Single‐Cell Analysis

Single‐cell RNA sequencing (scRNA‐seq) data from 28 glioma patients were obtained from the GEO dataset GSE131928, comprising a total of 24,131 cells for subsequent analysis. The original UMI count matrix was imported into R, and a Seurat object was constructed using the Seurat package to facilitate downstream analysis [28]. For quality control, we adhered to the processing methodology outlined in the original high‐quality publication. Specifically, the number of detected genes per cell exhibited a bimodal distribution; therefore, cells with fewer than 3000 detected genes were conservatively excluded, resulting in an average of approximately 5730 detected genes per cell. Concurrently, the mitochondrial transcript ratio was assessed using the PercentageFeatureSet function in the Seurat package to identify low‐quality or dead cells. Filtering was conducted based on several criteria, including the number of detected genes, total UMI count, and mitochondrial transcript ratio. Subsequently, the data normalisation process was executed using the NormalizeData function. Following this, the FindVariableFeatures function was employed to identify highly variable genes. For dimensionality reduction, the RunPCA function was applied to these highly variable genes, selecting the first 30 principal components for subsequent graph construction. A K‐nearest neighbours (KNN) graph was constructed using the FindNeighbors function, and cell clustering was performed with the FindClusters function, utilising a resolution parameter of 0.65. The clustering outcomes were visualised in two dimensions via the RunUMAP function. To identify marker genes for each cluster, differential expression analysis was conducted using the FindAllMarkers function, comparing the target cluster with the remaining cells. Finally, cell type annotation was primarily based on the GBmap reference, wherein the marker genes of each cluster were compared with the cell type characteristics delineated in the GBmap to complete the annotation process.

## 3 Results

### 3.1 Pan‐Cancer Analysis Revealed That ALG1 Was Highly Expressed in Most Tumours and Was Associated With a Poor Prognosis

**FIGURE 1:**
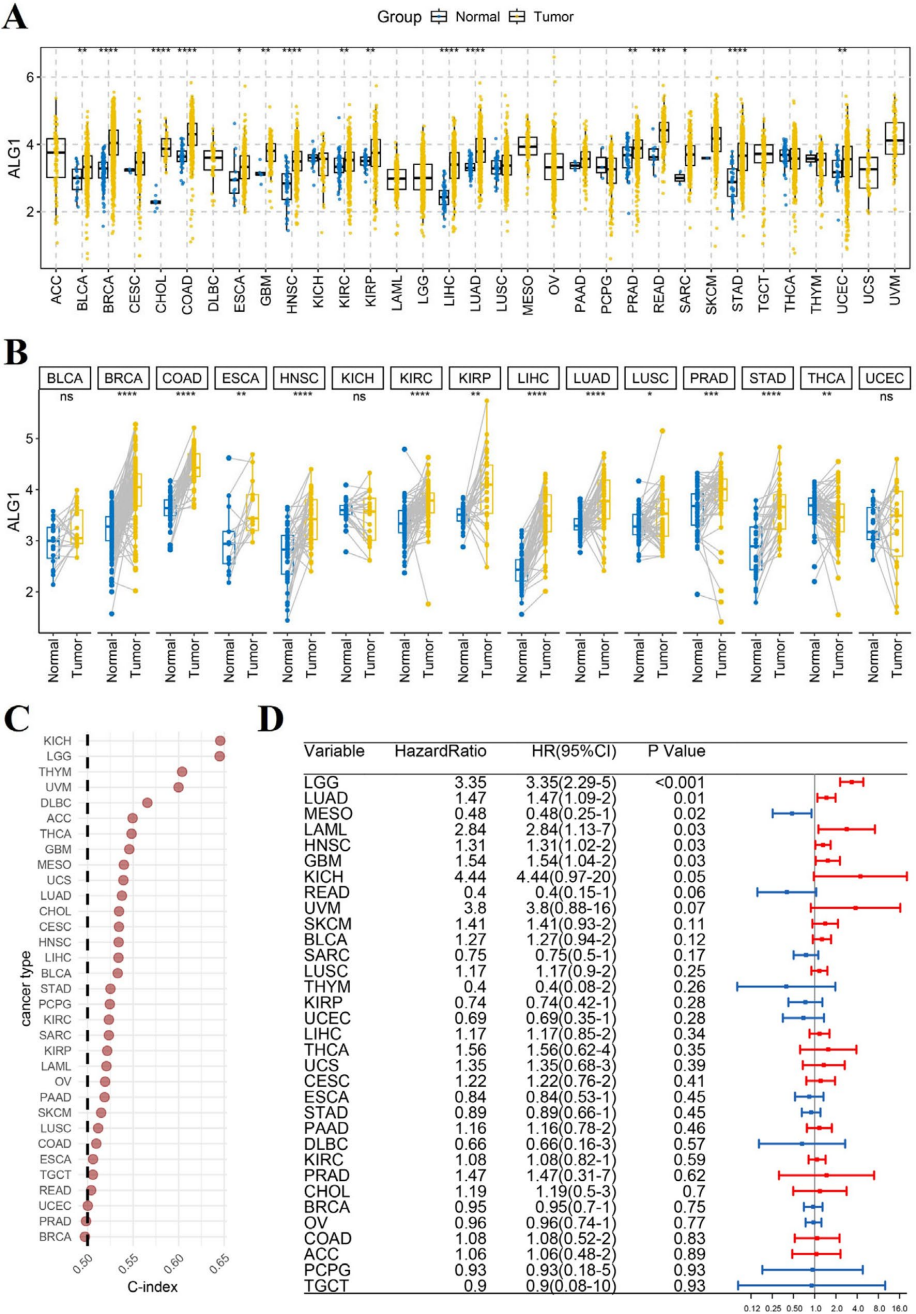
Pan‐cancer analysis of ALG1 expression. (A) Differential expression analysis of ALG1 across 33 tumour types in the TCGA cohort. ns indicates no statistical significance; *p < 0.05, **p < 0.01, ***p < 0.001, respectively (unpaired Wilcoxon test); (B) Paired differential expression analysis of ALG1 between tumour and matched normal tissues across 33 cancer types (paired Wilcoxon test); (C) Prognostic significance of ALG1 expression in various cancers based on univariate Cox regression analysis; (D) Forest plot illustrating the association between ALG1 expression and overall survival across different tumour types.

We analysed the differential expression of ALG1 between tumour and normal tissues across various cancer types using R software. Unpaired and paired Wilcoxon rank‐sum and signed‐rank tests were applied accordingly to assess statistical significance. The results revealed that ALG1 was significantly upregulated in 16 tumour types, including GBM and head and neck squamous cell carcinoma (HNSC) (Figure 1A,B). To evaluate the prognostic value of ALG1, Cox proportional hazards regression models were constructed for each cancer type. ALG1 expression has notable prognostic value for multiple tumour types, as concordance indices (C‐index) exceeded 0.5 for LGG, GBM, uveal melanoma (UVM), and other malignancies (Figure 1C). Furthermore, a forest plot analysis revealed that elevated ALG1 expression was associated with an increased risk of mortality in 20 cancer types, including LGG, GBM, and kidney chromophobe (KICH) (Figure 1D).

### 3.2 Inclusion of ALG1 Could Improve the Predictive Model

**FIGURE 2:**
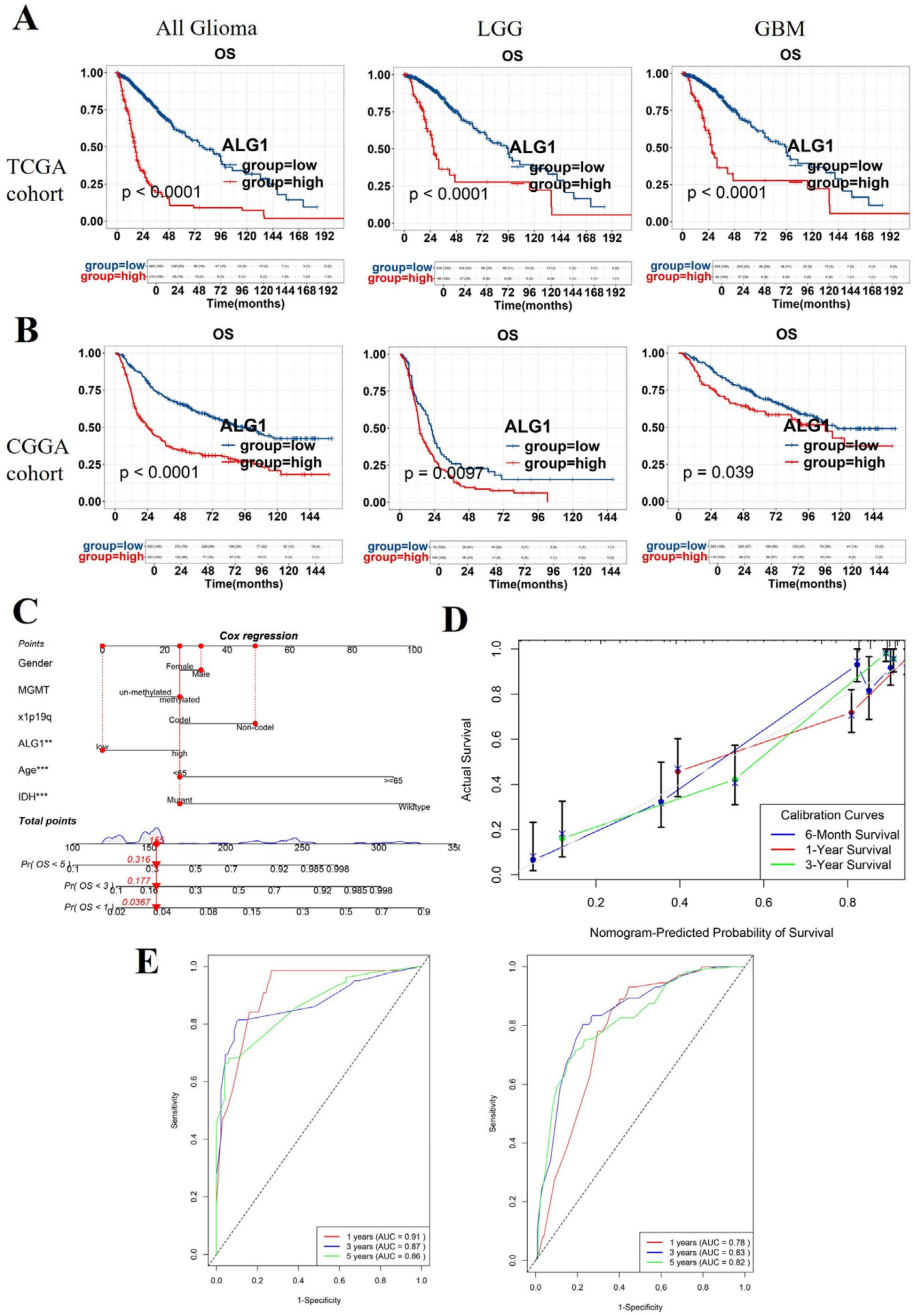
Construction and validation of ALG1 expression as a prognostic indicator. (A) Kaplan–Meier overall survival curves for patients with LGG, GBM, and all gliomas in the TCGA cohort and CGGA cohort, respectively; (B) Nomogram predicting 1‐, 3‐, and 5‐year overall survival probabilities in glioma patients based on age, WHO grade, IDH status, 1p/19q codeletion status, MGMT promoter status, and ALG1 expression. (C) Calibration plots evaluating the agreement between the predicted and observed survival outcomes (the grey dashed line represents the actual survival curve); (D, E) Time‐dependent ROC curves assessing the predictive accuracy of the nomogram model for 1‐, 3‐, and 5‐year overall survival. The results are shown for the TCGA training set (left) and the CGGA validation set (right).

To investigate the impact of ALG1 expression on glioma patient survival. Kaplan–Meier survival analyses revealed that patients with high ALG1 expression exhibited significantly poorer OS than did those with low ALG1 expression in the combined glioma cohort, and the same was observed for those in the LGG and GBM subgroups within both the TCGA and CGGA datasets (Figure 2A). Nomograms are valuable clinical tools that integrate multiple prognostic variables to estimate individual risk [29]. We constructed a nomogram that incorporates age, WHO grade, IDH status, 1p/19q status, the MGMT status and the expression of ALG1 to predict the 1‐, 3‐, and 5‐year OS probabilities. Each variable was assigned a specific score, and total scores were calculated to estimate individual survival probabilities. For example, a patient with a total score of 115 was predicted to have a 1‐year OS of 37.6%, a 3‐year OS of 17.7%, and a 5‐year OS of 3.67% (Figure 2B). Calibration curves were used to assess the accuracy of the nomogram in predicting survival among patients with high ALG1 expression. The predicted survival rates closely matched the observed outcomes at 1‐, 3‐, and 5‐years (Figure 2C). Time‐dependent ROC curves further demonstrated the discriminative power of the nomogram. In the TCGA training cohort, the model achieved area under the curve (AUC) values of 0.78, 0.83, and 0.82 for 1‐, 3‐, and 5‐year OS, respectively, and a similar predictive performance was validated in the CGGA validation cohort (Figure 2D,E).

### 3.3 High ALG1 Expression Was Associated With Poor Prognosis

**FIGURE 3:**
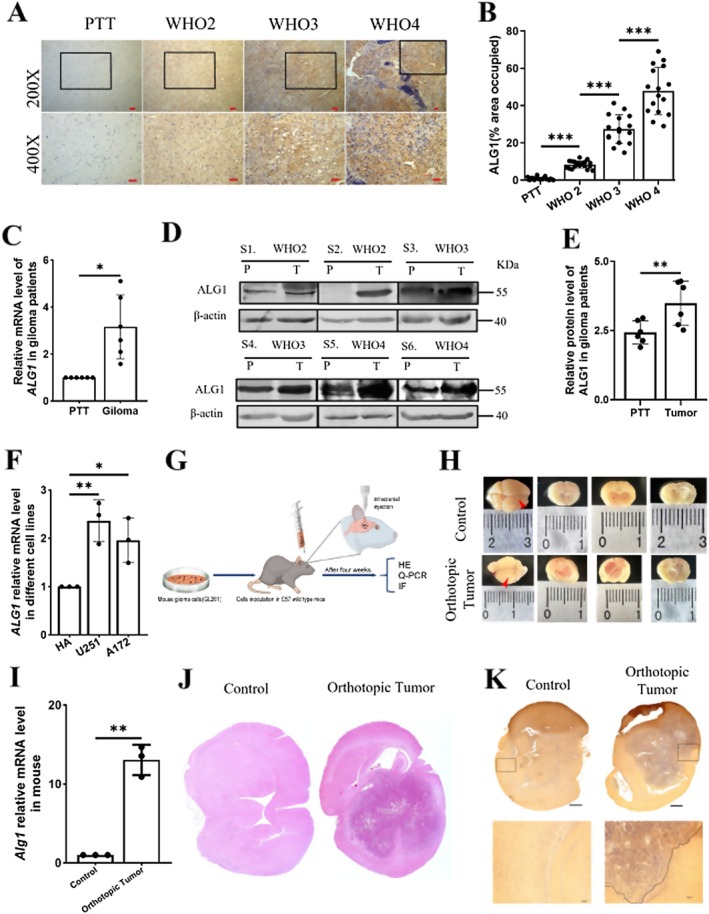
Validation of ALG1 expression in glioma samples, cell lines and intracranial model.(A, B) IHC staining and quantification of ALG1 in PTTs and glioma samples from 52 patients of different WHO grades (original magnifications: 200× and 400×), analysed using one‐way ANOVA; (C, D) RT‐qPCR and WB analysis of *ALG1* mRNA expression and ALG1 protein levels in paired PTTs and glioma samples from six patients, analysed using paired t tests; (E) Densitometric analysis of ALG1 protein expression via WB, as evaluated by paired t tests; (F) RT‐qPCR analysis of ALG1 mRNA expression in different cell lines (HAs, U251, and A172), assessed using one‐way ANOVA; (G, H) Schematic diagram of intracranial model development and comparing the whole brain and sections between the experimental group and the control group (red arrow represents the injection site); (I) RT‐qPCR analysis of ALG1 mRNA expression between the experimental group and the control group in intracranial model, assessed using paired t tests; (J) HE staining images of orthotopic tumour sections confirmed the tumour model successfully construction. Scale bars, 1.00 mm; (K) IHC staining of ALG1 in control brain tissue and experimental group in the intracranial model. The central region represents the magnified portion of the black square. Scale bars, 1.00 mm. *p < 0.05, **p < 0.01, ***p < 0.001.

We investigated the associations between ALG1 expression and glioma prognosis at both the tissue and cellular levels. IHC analysis of glioma tissues with different WHO grades revealed a significant positive correlation between ALG1 expression and tumour grade. In contrast, ALG1 expression was nearly undetectable in brain tissues from healthy individuals (Figure 3A,B). ALG1 expression had a significant upregulation at both the mRNA and protein levels in six glioma tissue samples and paired peritumoral tissues (PTTs) (Figure 3C–E). Additionally, RT‐qPCR analysis of ALG1 mRNA expression revealed that ALG1 expression was markedly higher in glioma cells (U251 and A172) than in normal human microglia (HA) (Figure 3F). Furthermore, an orthotopic tumour model was successfully established in C57BL/6 mice via stereotactic intracranial injection of murine‐derived GL261 cells (Figure 3G,H). Four weeks post‐inoculation, brain tissues were harvested for gross morphological observation and H&E staining to confirm tumour formation (Figure 3J). Subsequent assessment of ALG1 expression in tumour‐bearing mice revealed a significant upregulation of ALG1 mRNA in the experimental group by RT‐qPCR (Figure 3I), while IHC staining demonstrated markedly elevated ALG1 protein expression localised within the tumour regions (Figure 3K).

### 3.4 Potential Biological Functions and Pathways of ALG1 in Glioma Progression

**FIGURE 4:**
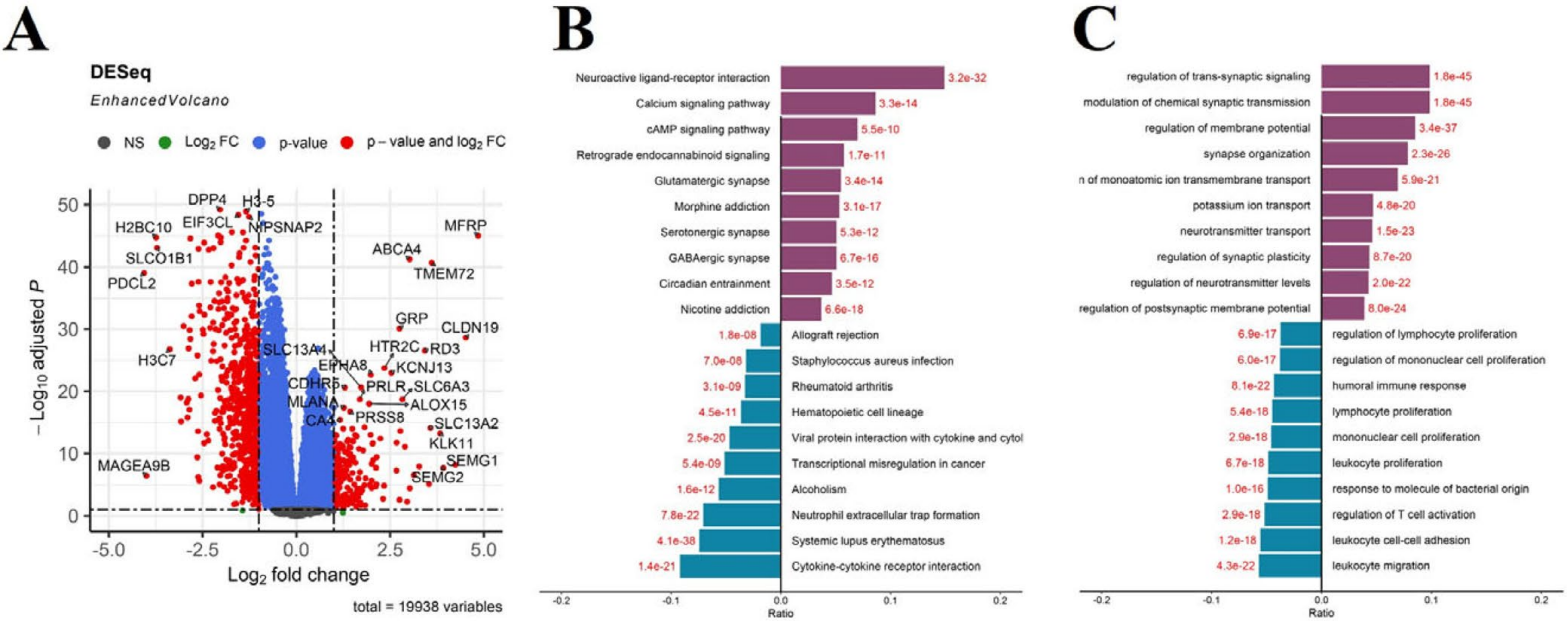
Gene enrichment analysis of ALG1‐associated biological functions in glioma. (A) Volcano plot showing DEGs between the high and low ALG1 expression groups in the TCGA cohort; (B, C) GO and KEGG enrichment analysis of DEGs highlighting key biological processes related to ALG1 expression.

To elucidate the potential mechanisms by which ALG1 contributes to glioma development, we identified a total of 1861 DEGs between the high and low ALG1 expression groups. Among these genes, 731 were upregulated, and 1130 were downregulated (Figure 4A). GO functional annotation and KEGG pathway enrichment analyses were subsequently performed on the identified DEGs. GO analysis revealed that the DEGs were predominantly enriched in processes such as regulation of transsynapticsignalling, modulation of chemical synaptic transmission, and regulation of membrane potential (Figure 4B). KEGG pathway analysis further revealed that the DEGs were involved mainly in the neuroactive ligand‐receptor interaction, cytokine‐cytokine receptor interaction, and calcium and cAMP signalling pathways (Figure 4C).

### 3.5 ALG1 Expression Was Associated With Immune Cell Infiltration in the TME of Gliomas

**FIGURE 5:**
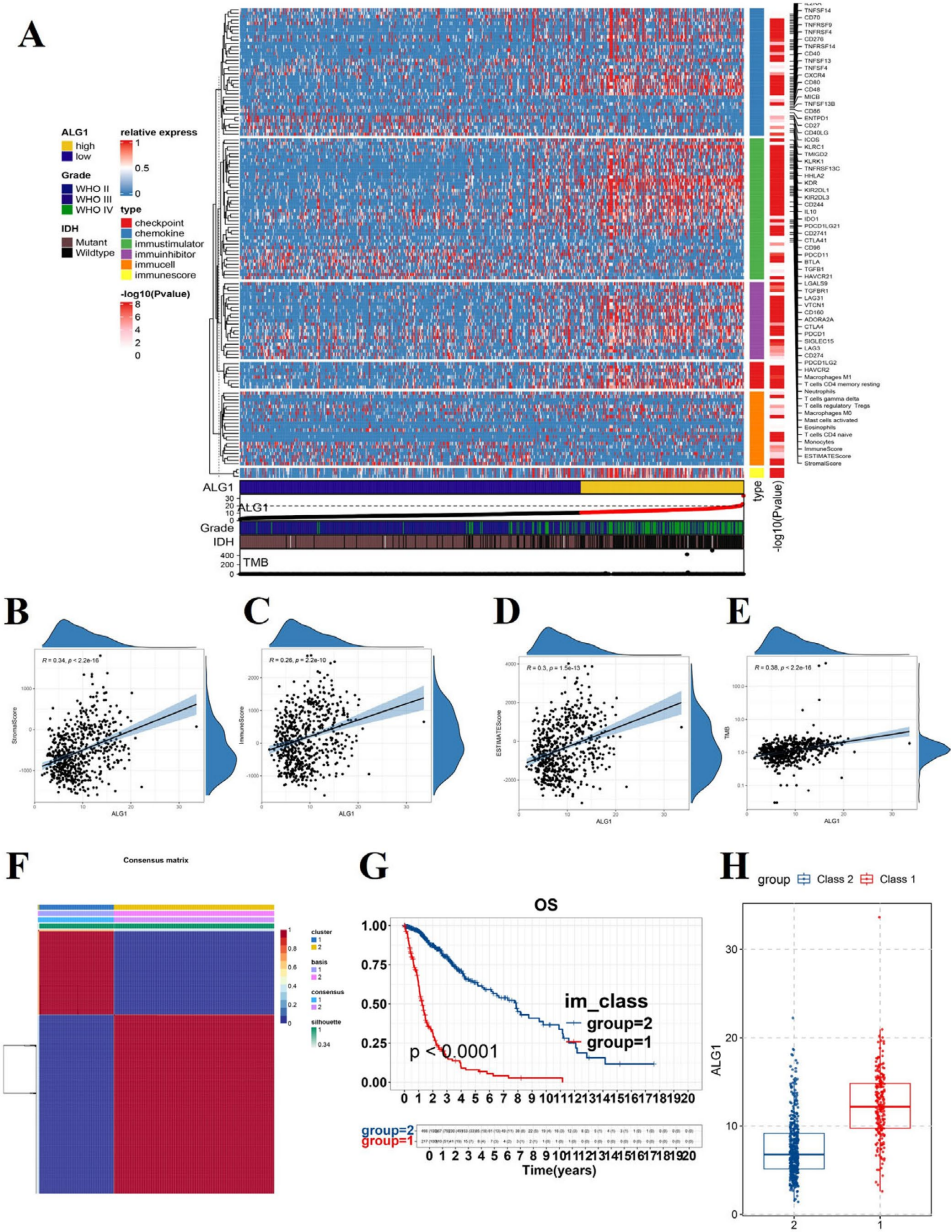
Association between ALG1 expression and the tumour immune microenvironment in glioma. (A) Heatmap showing the relative expression levels of various immune‐related genes in glioma patients with high ALG1 expression; (B–E) Scatter plots illustrating the Spearman correlation between ALG1 expression and the stromal score, immune score, ESTIMATE score, and TMB; (F) Clustering of glioma samples into two subtypes (C1 and C2) based on cophenetic correlation values and the expression profiles of immune‐related genes; (G) Kaplan–Meier survival curves comparing the overall survival between patients in the C1 group and those in the C2 group; (H) Comparison of ALG1 expression levels between the C1 and C2 clusters; *p* < 0.001.

To investigate the relationship between ALG1 expression and the tumour immune microenvironment, we generated a heatmap displaying the top 100 genes positively or negatively correlated with ALG1 expression. The heatmap indicated that patients with high ALG1 expression exhibited elevated immune cell infiltration (Figure 5A). Furthermore, ALG1 expression was significantly correlated with several TME parameters: The stromal score, immune score, ESTIMATE score, and tumour mutational burden (TMB) (Figure 5B–E). We further explored the associations between ALG1 expression and various infiltrating immune and stromal cell types. Correlation analysis showed that ALG1 expression was negatively correlated with infiltrating B cells. In contrast, ALG1 expression was positively correlated with CD8^+^ T cells, NK cells, CAFs, and macrophages. Scatter plots illustrating these correlations are shown in Figure S1. We next downloaded 2483 immune‐related genes and conducted NMF clustering. According to cophenetic correlation coefficients, glioma samples were stratified into two immune subtypes: C1 and C2 (Figure 5F). Survival analysis revealed that patients in the C2 group had significantly worse outcomes than the C1 group (Figure 5G). Notably, ALG1 expression was significantly higher in the C2 subtype than in the C1 subtype (Figure 5H). To further investigate these findings, we conducted co‐staining of ALG1, CD8, CD4, and PD‐L1 in glioma tissue sections across various WHO grades. Consistent with our bioinformatic observations, the co‐expression of ALG1 with CD4, CD8, and PD‐L1 progressively increased with higher tumour grades (Figure S2). These histological findings are in accordance with the correlation between ALG1 expression and immune infiltration observed in the TCGA database.

### 3.6 Identification of Dynamic ALG1 Genes During the Transition of Cell States

**FIGURE 6:**
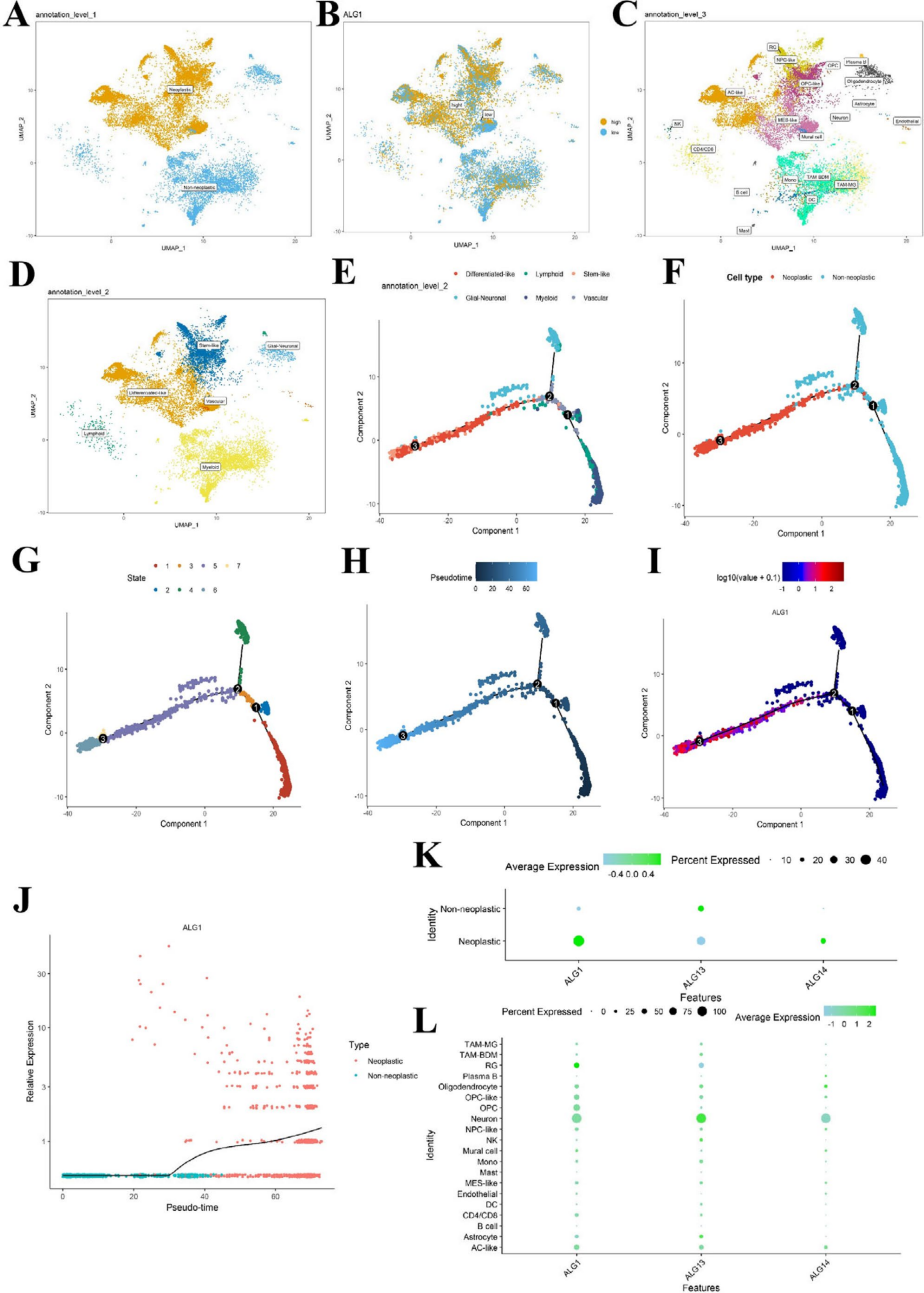
Clustering of single‐cell RNA sequencing data and expression dynamics of ALG1 during glioma progression. (A) UMAP plot showing the cellular composition of single‐cell transcriptomic data; (B) Expression levels of ALG1 across different cell type populations; (C) Malignant glioblastoma cell states, including AC‐like, NPC‐like, OPC‐like, and MES‐like states, and immune‐related marker expression mapped across the main cellular subclusters; (D) Distribution of cell subsets of differentiated, lymphoid, stem, glial‐neuronal, myeloid, and vascular cells; (E) Annotation of cell types, including differentiated‐like, lymphoid, stem‐like, glial‐neuronal, myeloid, and vascular lineages; (F) Distribution of tumour and nontumor cells along pseudotime trajectories; (G) Developmental trajectories of single cells inferred using pseudotime analysis; (H) Pseudotime trajectories distinguishing nontumor cell from tumour cell populations; (I) Expression dynamics of ALG1 along the pseudotime axis; (J) Feature plot showing the temporal expression pattern of ALG1 during gliomagenesis; (K, L) Bubble chart showing that ALG1 is highly expressed in both malignant cells and differentiated‐like cells.

The scRNA‐seq technology provides insights into the transcriptional regulation and dynamic evolution of individual cells in healthy human brains and in malignant tumours [30]. To investigate whether ALG1 contributes to malignant cell fate determination during glioma initiation and progression, we performed single‐cell trajectory analysis. All cells were categorised into neoplastic and non‐neoplastic populations based on their expression signatures. We found ALG1 expression to be heterogeneous across cell types but predominantly elevated in neoplastic cells (Figure 6A,B). Moreover, ALG1 was predominantly expressed in AC‐like, NPC‐like, OPC‐like, and MES‐like cellular states, which are known to drive the malignant cellular heterogeneity of glioblastoma [31] (Figure 6C). Interestingly, ALG1 was also highly expressed in both differentiated‐like and stem‐like cellular states, and the cells were further annotated into six major lineages: Differentiated‐like, lymphoid, stem‐like, glial‐neuronal, myeloid, and vascular (Figure 6D,E). To examine the dynamic changes in ALG1 expression during the transformation from non‐neoplastic to neoplastic states, pseudotime trajectory analysis was performed (Figure 6F). According to the inferred developmental trajectory, the cells were classified into seven distinct states (Figure 6G), and Root 6, corresponding to the distribution of non‐neoplastic cells, was selected as the starting point for the pseudotime timeline of ALG1 expression (Figure 6H,I). As shown in Figure 6J, ALG1 expression gradually increased throughout the progression from a nontumor state to a malignant state. Moreover, a bubble plot analysis demonstrated that, compared with other ALG family members, such as ALG13 and ALG14, ALG1 exhibited markedly higher expression in malignant and differentiated‐like cellular states (Figure 6K,L), which supports its protumorigenic role in glioma progression and cellular transformation.

### 3.7 ALG1 Expression Is Associated With Intercellular Communication and Signalling in Glioma Cells

**FIGURE 7:**
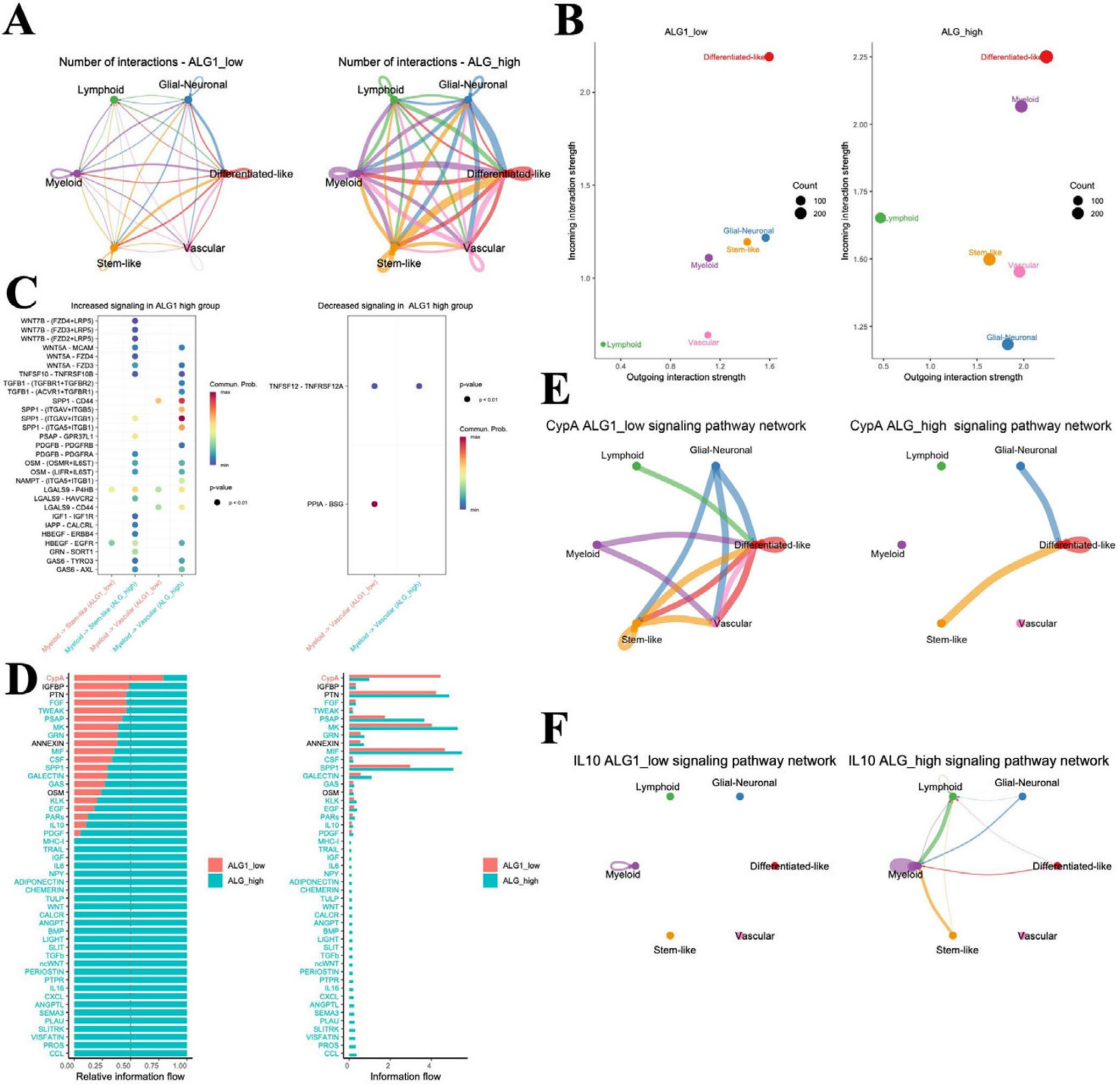
Single‐cell intercellular communication analysis under different ALG1 expression states in glioma. (A) Cell–cell interaction network depicting the number and strength of signalling interactions among various glioma cell states under high and low ALG1 expression conditions; (B) Quantitative assessment of incoming and outgoing signalling strength across cell populations. The circle size indicates overall communication strength; (C) Bubble plot showing the probability and significance of ligand–receptor interactions in the ALG1‐high and ALG1‐low groups. The circle size corresponds to the *p*‐value, and the red intensity indicates the likelihood of interaction; (D) Identification and visualisation of differentially enriched signalling pathways between the ALG1‐high‐ and ALG1‐low‐expression groups. Pathways enriched in the low‐expression group are shown in red (top), whereas those enriched in the high‐expression group are shown in green (bottom); (E, F) Comparative analysis of the CypA (E) and IL10 (F) signalling pathways under different ALG1 expression levels, illustrating their communication dynamics across distinct cell lineages.

CellChat, a recently developed machine learning framework, enables quantitative inference of intercellular communication networks [32]. A cell–cell interaction network analysis based on ALG1 expression showed that the high ALG1 expression group exhibited increased quantity and strength of intercellular signalling among different glioma cell states. Quantitative assessment of outgoing and incoming signalling strength indicated that differentiated‐like cells were key signalling hubs in the ALG1 high‐expression group (Figure 7A,B). A bubble plot analysis further demonstrated a notable increase in both the number and intensity of ligand–receptor interactions in the ALG1‐high group (Figure 7C, left), whereas only limited communication events were observed in the ALG1‐low group (Figure 7C, right). Signalling pathway enrichment analysis revealed that ALG1 expression was associated with substantial alterations in intercellular communication networks. Compared with the ALG1‐low group, the ALG1‐high group exhibited increased signalling activity in multiple pathways, including FGF, MK, MIF, and SPP1signalling. In contrast, pathways such as CypA and IGFBP were more active in the ALG1‐low group (Figure 7D). These findings suggest that elevated ALG1 expression reshapes the intercellular signalling landscape in glioma and may contribute to tumour progression and immune microenvironment modulation. Analysis of the CypA signalling pathway showed frequent interactions between differentiated‐like cells and other cell subsets in the low‐ALG1 expression group, whereas in the ALG1‐high group, communication was concentrated on differentiated‐like cells to stem‐like and glial‐neuronal subsets (Figure 7E). Similarly, IL10 signalling patterns differed with ALG1 expression. In the high‐expression group, myeloid cells emerged as the main source of IL10signalling, targeting both differentiated‐like and lymphoid cell populations (Figure 7F). Collectively, these findings suggest that ALG1 expression is associated with changes in glioma intercellular communication networks, with differentiated‐like cells as central mediators in immune‐relatedsignalling.

### 3.8 ALG1 Knockdown Inhibits the Migration of Glioma Cells in Vitro

**FIGURE 8:**
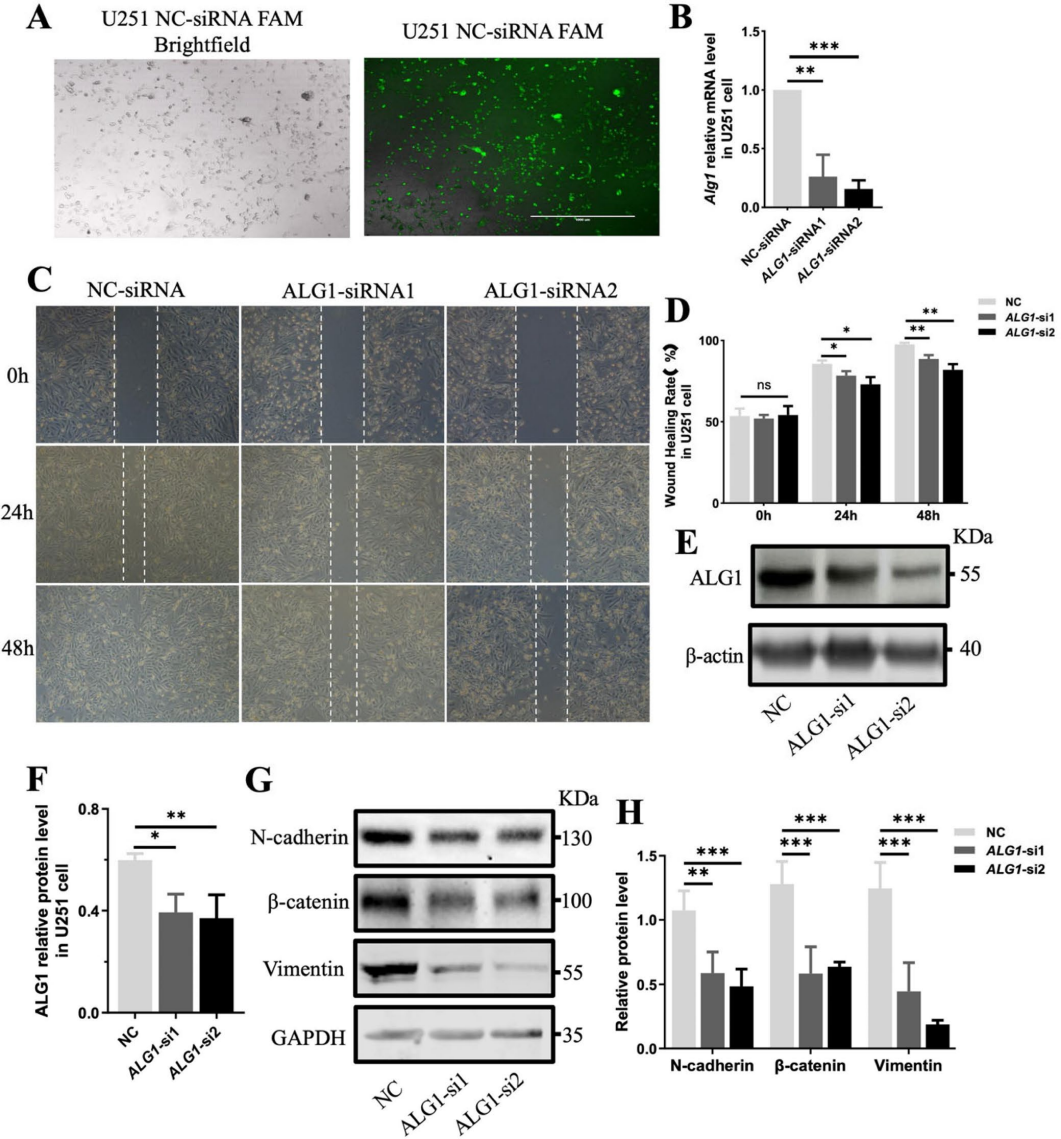
Functional role of ALG1 in glioma cell migration and proliferation. (A) Evaluation of siRNA transfection efficiency in U251 cells using FAM‐labelled NC‐siRNA; (B) Knockdown efficiency of ALG1 mRNA in U251 cells following transfection with ALG1‐targeting siRNA, as assessed by RT‐qPCR; (C, D) Wound healing assays performed at 0 h, 24 h, and 48 h after ALG1 knockdown and quantification of the percentage of wound closure in scratch assays; (E, F) WB analysis confirming ALG1 knockdown at the protein level and corresponding densitometric quantification; (G, H) Expression levels of N‐cadherin, β‐catenin, and vimentin after ALG1 knockdown, with GAPDH or β‐Actin as the loading control. Densitometric analysis was conducted for quantitative comparison. All the data are presented as the means ± standard deviations (SDs) from at least three independent experiments. Statistical comparisons were performed using unpaired *t* tests or one‐way ANOVA, as appropriate. *p < 0.05, **p < 0.01, ***p < 0.001.

To further investigate the functional role of ALG1 in glioma cells, we first assessed the transfection efficiency of the FAM‐labelled NC siRNA. The results demonstrated that the transfection efficiency in the U251 glioma cell line exceeded 95% (Figure 8A). Two targeted ALG1‐siRNAs were subsequently transfected into U251 cells to knock down ALG1 expression, with NC‐siRNA serving as a negative control. ALG1 expression was significantly lower at both the mRNA and protein levels following ALG1‐siRNA transfection (Figure 8B,E,F). Wound healing assays further demonstrated that ALG1 knockdown markedly inhibited the migration of U251 cells (Figure 8C,D). Similarly, we obtained the same result in another glioma cell line, A172 (Figure S3). In addition, we examined the expression levels of N‐cadherin, β‐catenin, and vimentin. The results revealed that silencing ALG1 led to downregulation of these markers (Figure 8G,H), which indicates that reduced ALG1 expression suppresses glioma cell migration.

## 4 Discussion

Glioma is a fatal tumour of the human CNS characterised by high malignancy, and none of the current treatment strategies have achieved satisfactory clinical efficacy [33]. The overall prognosis of GBM has a 5‐year survival rate of less than 10% [34, 35]. Therefore, the development of effective biomarkers for diagnosis and prognosis is crucial. Studies have shown that treatment failure in cancer is often attributed to late‐stage diagnosis, tumour recurrence, and metastasis, all of which are closely associated with glycosylation [15]. Numerous studies in glycomics and glycoproteomics have established a strong link between aberrant glycosylation and glioma pathogenesis [36]. ALG1 contributes to the early stages of protein glycosylation [37, 38]. Previous studies have shown that ALG1 is involved in the glycosylation of N‐cadherin and plays a critical role in promoting HCC migration [18]. However, little is known about the clinical implications and biological functions of ALG1 in glioma.

This study is the first to comprehensively analyse the expression and clinical significance of ALG1 in glioma. Univariate Cox regression analysis identified ALG1 as an independent prognostic factor for glioma. Kaplan–Meier survival analyses revealed that high ALG1 expression was significantly associated with reduced OS in glioma patients, which suggests that ALG1 may serve as a valuable prognostic biomarker. We further constructed a nomogram to calculate a total score for estimating 1‐, 3‐, and 5‐year OS in glioma patients based on unfavourable prognostic variables. The calibration curves confirmed the model predictive accuracy for glioma patients with high ALG1 expression. IHC staining confirmed the association between ALG1 expression and WHO tumour grade and revealed a stepwise increase in ALG1 expression with increasing glioma grade. Moreover, RT‐qPCR and WB revealed elevated ALG1 expression in glioma tissues, which is consistent with glioma cell lines and the orthotopic tumour mouse model. Interestingly, these findings contrast with previous reports for HCC, in which reduced ALG1 expression was associated with tumour metastasis and poor survival [39]. This discrepancy suggests that ALG1 may play dual roles in different cancers. Similar dual functionality has been reported for other glycosyltransferases, such as MGAT3 and FUT8 [40, 41]. To explore potential mechanisms, GO and KEGG enrichment analyses were performed and revealed that high ALG1 expression was significantly associated with immune‐related pathways. Previous studies have shown that overactivation of glutamate signalling in the neuroactive ligand‐receptor pathway and calcium‐dependent cascades can increase cellular excitability and promote GBM invasion [42]. Similarly, impaired N‐glycosylation has been shown to suppress AKT activation and downstream cAMP/PKAsignalling, which contributes to increased glioma invasiveness [43]. Collectively, these findings suggest that ALG1 may influence glioma prognosis by influencing these signalling pathways.

Previous studies have demonstrated that immune cell infiltration and activation play crucial roles in glioma progression and the response to immunotherapy [44, 45]. However, the role of ALG1 within the TME has not been previously reported. In this study, we report for the first time that high ALG1 expression is significantly associated with upregulation of multiple immune checkpoint molecules and strongly correlated with the immune score, stromal score, TMB, and tumour purity, suggesting its potential involvement in immune evasion and the TME. Moreover, ALG1 expression is positively correlated with CD4^+^ T cells and CD8^+^ T cells. Notably, CD4^+^ and CD8^+^ T cells have been recognised as prognostic indicators in glioma [46]. We also observed increased co‐expression of ALG1 with PD‐L1, which highlights its potential as a novel immunotherapeutic target. Importantly, inhibition of ALG3, a glycosyltransferase involved in N‐glycosylation, has been shown to increase the efficacy of immune checkpoint blockade therapy, underscoring the critical role of glycosyltransferases in the immune microenvironment [47]. As a member of the ALG family, ALG1 may influence glioma prognosis by modulating immune cell infiltration.

The dynamic expression pattern of ALG1 observed during glioma cell state transitions highlights its potential regulatory role in gliomagenesis. In our study, single‐cell trajectory analysis revealed that ALG1 expression was markedly upregulated in neoplastic cells and progressively increased along the pseudotime axis during the transition from non‐neoplastic to neoplastic states. This suggests that ALG1 may contribute to glioma initiation by promoting early tumour cell activation. Notably, ALG1 was highly expressed across all malignant cell states, which supports its potential role in glioma. Advancements in bioinformatics have enabled comprehensive visualisation of intercellular communication across diverse cellular subpopulations. In this study, we revealed a complex cell–cell communication network between differentiated‐like cells and other cellular states under various ALG1 expression levels, which suggests a potential role for ALG1 in supporting both tumour cell differentiation. Pathway‐specific enrichment analysis revealed that differential ALG1 expression significantly reshaped the cellsignalling, particularly through modulation of the CypA and IL10 signalling pathways. Previous research has demonstrated that CypA is markedly overexpressed in aggressive gliomas, where it promotes tumour progression via its receptor CD147 and activation of downstream pathways, including the MAPK/ERK, PI3K/AKT, STAT3, and Wnt/β‐catenin pathways [48, 49]. IL10, a pivotal immunosuppressive cytokine within the glioma TME, promotes immune evasion by upregulating PD‐L1 expression, activating STAT3signalling, and inducing T‐cell exhaustion [50, 51]. Therefore, future studies should investigate whether ALG1 modulates these critical signalling pathways to drive gliomagenesis.

Finally, we confirmed the functional role of ALG1 in glioma progression using in vitro assays. ALG1 knockdown in U251 glioma cells reduced cell migration. WB analysis revealed reduced expression of β‐catenin, N‐cadherin, and vimentin upon ALG1 knockdown, which suggests a tumour‐promoting role for ALG1 in glioma. β‐catenin, a key component of the Wnt signalling pathway, is frequently associated with enhanced tumorigenesis and metastasis [52, 53]. N‐cadherin, a cell adhesion molecule, is typically upregulated in tumours and facilitates decreased intercellular adhesion and increased invasiveness [53]. Vimentin, a marker of mesenchymal transition, is often linked to tumour metastasis and dedifferentiation [54]. Collectively, these findings indicate that ALG1 plays a critical role in regulating glioma cell migration and invasion.

This study has several limitations. First, we integrated robust open‐access datasets and in vitro experiments to demonstrate the oncogenic role of ALG1 in glioma for the first time; however, the underlying molecular mechanisms require further elucidation. Although our study demonstrated that ALG1 knockdown significantly inhibited glioma cell migration and reduced the expression of EMT‐related proteins, cell proliferation assays such as CCK‐8 or EdU incorporation were not performed in the present study. Therefore, the potential effect of ALG1 on glioma cell proliferation requires further investigation. Future studies will focus on performing proliferation assays and rescue experiments to validate the functional role of ALG1 more comprehensively. Second, TCGA datasets have some disadvantages, such as bias in glioma types, which may introduce inevitable selection biases. Third, and importantly, the relationship between ALG1 and immune infiltration was established through computational analyses, and only IF staining was used to verify the correlation between some immune factors and ALG1. No additional in‐depth typing of immune molecules or in vivo verification using animal models with ALG1 knockdown was performed. Nevertheless, by leveraging the TCGA database and functional assays, in this study, the expression of ALG1 and its impact on glioma malignancy, as well as its correlation with clinicopathological features, prognosis, immune infiltration, and single‐cell analysis, were systematically investigated. These findings provide novel insights into the potential role of ALG1 in gliomagenesis and tumour immunoregulation.

## Author Contributions

Shuxiang Li, Yang Xia and Qiang Liu designed and performed the experiments. Qian Xin and Anhong Liu embed and cut the paraffin blocks of mouse brain tissues. Jia Wang and Xinyu Wang helped in collecting the clinical samples and data. Peng Gao and Di Zuo evaluated the amount and quality of the tumor tissues. Jing Zhang, Jinhai Ma and Peng Gao conceived and supervised the project. Shuxiang Li and Jing Zhang wrote the manuscript.

## Funding

This work was supported by the Natural Science Foundation of Ningxia (2024AAC03620, 2024AAC03554, 2024AAC03590, 2023AAC03600); the Scientific Research Program of Ningxia Colleges and Universities (NYG2024129); the Ningxia Medical University scientific research project (LNKF202301); and the College Students’ Innovative Entrepreneurial Training Plan Program (S202210752030).

## Ethics Statement

The study received ethical approval from the Medical Research Ethics Review Committee of the General Hospital of Ningxia Medical University, under the approval number KYLL‐2025‐2249.

## Consent

All authors have given consent to publish.

## Conflicts of Interest

The authors declare no conflicts of interest.

## Supporting information


**Figure S1:** The relationship between the expression of ALG1 and immune cells was analysed by EPIC and TIMER.
**Figure S2:** Colocalisation of ALG1 with various immune markers in glioma tissues.
**Figure S3:** Functional role of ALG1 in glioma cells of A172.

**Table S1:** Summary of Clinicopathological Characteristics of Glioma Patients.
**Table S2:** Clinical Data of Glioma Patients.

## Data Availability

The TCGA data and CGGA data that support the findings of this study are openly available at http://cancergenome.nih.gov and http://www.cgga.org.cn/, respectively.
