# Supplementary material for: High ALG1 Expression Is Correlated With Poor Prognosis and the Immune Microenvironment in Glioma
Source: J Cell Mol Med. 2026 Apr 19;30(8):e71142. doi: 10.1111/jcmm.71142 (PMC13092508; doi:10.1111/jcmm.71142)
Supplement: Supplementary file 1 — Figure S1: The relationship between the expression of ALG1 and immune cells was analysed by EPIC and TIMER. Figure S2: Colocalisation of ALG1 with various immune markers in glioma tissues. Figure S3: Functional role of ALG1 in glioma cells of A172. [file JCMM-30-e71142-s002.docx]

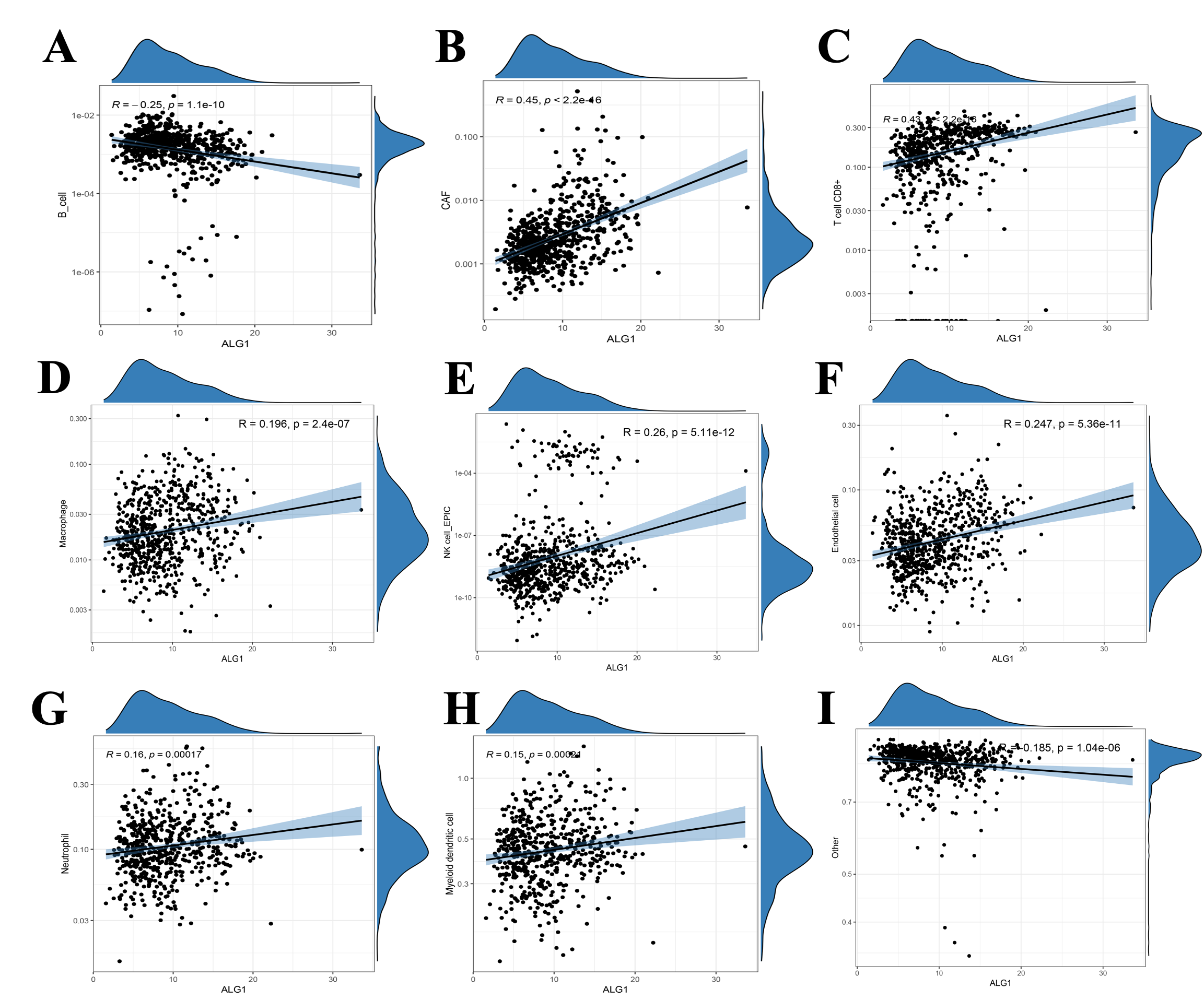


**Supplementary Figure 1 The relationship between the expression of ALG1 and immune cells were analyzed by EPIC and TIMER**. The scatter plot shows the correlation between ALG1 and B cells (A), CAFs (B), CD8^+^ T cells (C), macrophages (D), NK cells (E), Endothelial cells (F), Myeloid dendritic cells (G), Neutrophils(H) and other cells (I)


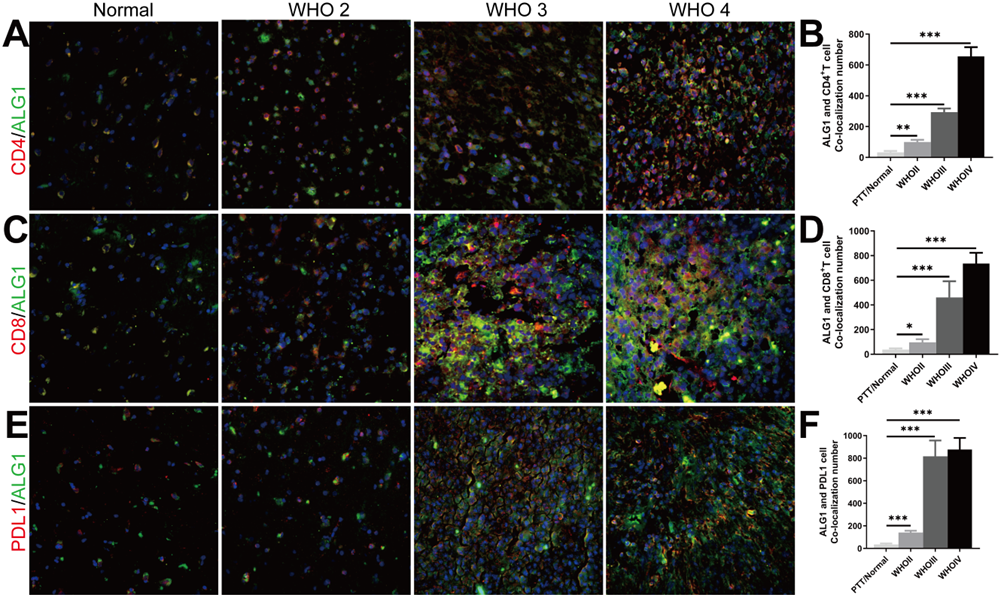


**Supplementary Figure 2 Colocalization of ALG1 with various immune markers in glioma tissues.** (A, C, E) Representative IF images showing the colocalization of ALG1 with CD4^+^ T cells (A), CD8^+^ T cells (C), and PD-L1 (E) in glioma tissues of different WHO grades. Sections were stained with primary antibodies against ALG1 (green) and the corresponding immune markers (red), and nuclei were counterstained with DAPI (blue). Images were captured at 400× magnification. (B, D, F) Quantification of colocalized cells corresponding to each marker shown in panels A, C, and E. Cell counts were obtained from five random high-power fields per section and analyzed using one-way ANOVA. *P*<0.05 was considered statistically significant.


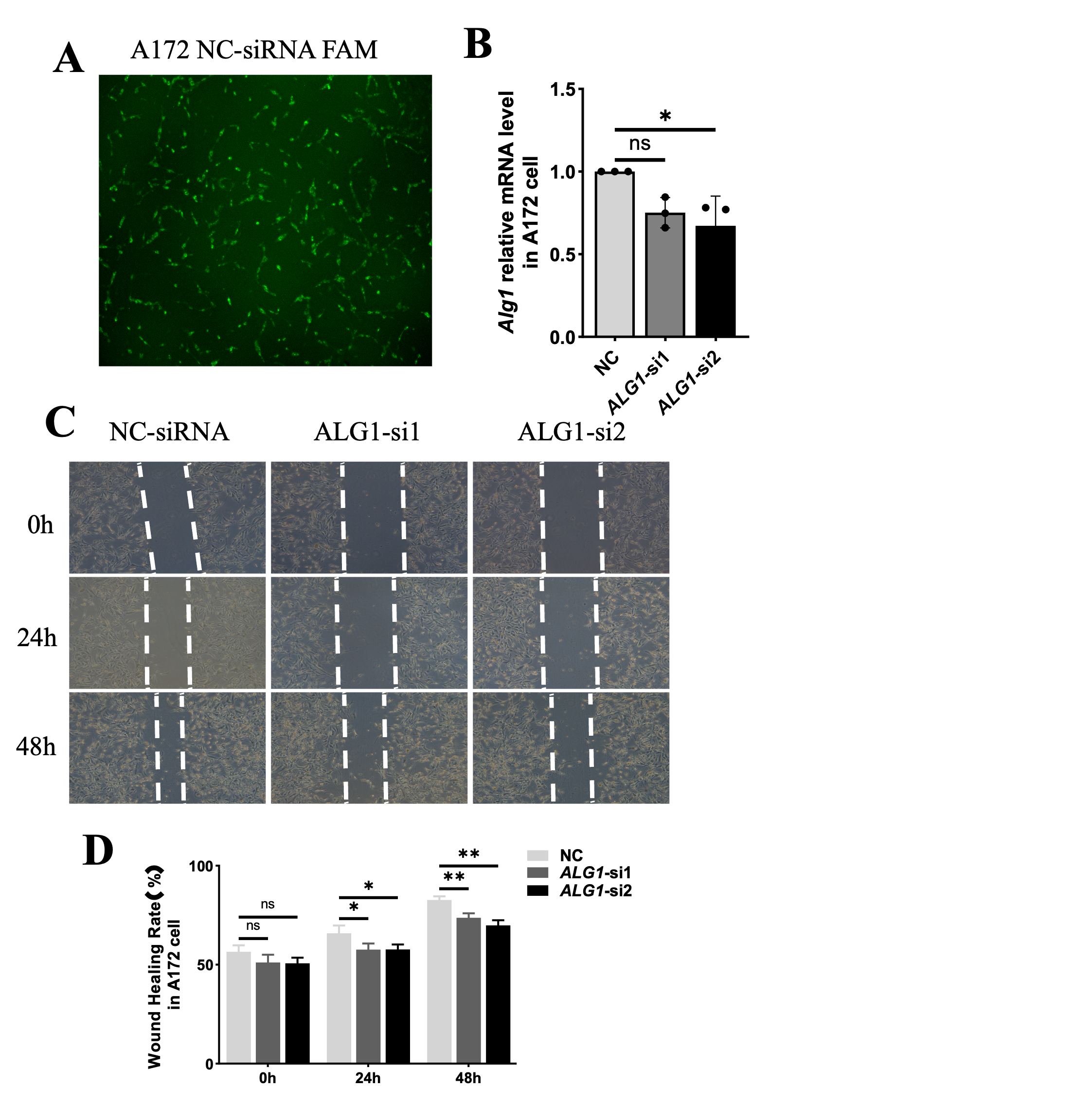


**Supplementary Figure 3 Functional role of ALG1 in glioma cell of A172.** (A) Evaluation of siRNA transfection efficiency in A172 glioma cells using FAM-labeled NC-siRNA. (B) Knockdown efficiency of ALG1 mRNA in A172 cells with ALG1-targeting siRNA by RT-qPCR. (C-D) Wound healing assays performed at 0 h, 24 h, and 48 h after ALG1 knockdown in A172 and Quantification of wound closure percentage by one-way ANOVA, *p* < 0.05 was considered significant.
