# Supplementary material for: High ALG1 Expression Is Correlated With Poor Prognosis and the Immune Microenvironment in Glioma
Source: J Cell Mol Med. 2026 Apr 19;30(8):e71142. doi: 10.1111/jcmm.71142 (PMC13092508; doi:10.1111/jcmm.71142)
Supplement: Supplementary file 2 — Table S1: Summary of Clinicopathological Characteristics of Glioma Patients. Table S2: Clinical Data of Glioma Patients. [file JCMM-30-e71142-s001.docx]

| Number |  | Gender | Age（years） | Diagnosis | WHO grade | Tumor size | Underlying medical conditions | IDH Mutation | MEMT modification | 1p19q co-deletion | P53 Mutation | others biomarkers |
| --- | --- | --- | --- | --- | --- | --- | --- | --- | --- | --- | --- | --- |
| Sample1 | PTT | Female | 69 | Intracranial space-occupying lesion (Diffuse astrocytoma) | WHO3 | 0.4cm，0.6cm | No | + | - | - | WT（-） |  |
|  | Tumor |  |  |  |  | PTT：5×4×2.8cm^3^ |  |  |  |  |  |  |
| Sample2 | PTT | Male | 52 | Intracranial space-occupying lesions (bilateral frontal lobes; Low-grade glioma | WHO2 | 0.5cm，0.4cm | No | + | + | + | + | Vim+，Olig2+，ATRX+，NF+，Syn+,P16+,S100+,GFAP+，EZH2+ |
|  | Tumor |  |  |  |  | PTT：7×4×1.5cm^3^ |  |  |  |  |  | (Ki67 10%),NeuN-,H3K27M- |
| Sample3 | PTT | Female | 54 | Intracranial space-occupying lesion (right basal ganglia; High-grade glioma | WHO4 | 0.5cm，0.5cm | No | - | - | - | + | Vim+，Olig2+，ATRX+，Syn+,P16+,S100+,GFAP+，EZH2+ |
|  | Tumor |  |  |  |  | PTT：5×4×2cm^3^ |  |  |  |  |  | (Ki67 60%),NeuN-,H3K27M-，NF- |
| Sample4 | PTT | Male | 72 | Intracranial malignant tumor (glioblastoma at the right frontal lobe-corpus callosum genu junction) | WHO4 | 0.4cm，0.7cm | No | - | + | - | + | Vim+，Olig2+，ATRX+，Syn+,P16+,S100+,GFAP+ |
|  | Tumor |  |  |  |  | PTT：5×4×2.8cm^3^ |  |  |  |  |  | (Ki67 20%),NeuN-,EZH2- |
| Sample5 | PTT | Male | 45 | Intracranial space-occupying lesion (high-grade diffuse astrocytoma) | WHO3 | 0.5×0.5×0.5cm，0.7cm | No | + | - | - | WT（-） | Vim+，Olig2+，ATRX+，Syn+,S100+,GFAP+，H3K27me3+ |
|  | Tumor |  |  |  |  | PTT：7×6×3cm^3^ |  |  |  |  |  | (Ki67 10%),NeuN-,EZH2-,H3K27M-，NF-,P16- |
| Sample6 | PTT | Male | 61 | Intracranial space-occupying lesion (diffuse glioma) | WHO2 | 0.4cm，0.4cm | hypertension, Hepatic hemangioma | + | - | + | WT（-） | Vim+，Olig2+，Syn+,S100+,GFAP+，H3K27me3+,NF+ |
|  | Tumor |  |  |  |  | PTT：3×2×1cm^3^ |  |  |  |  |  | (Ki67 2%),NeuN-,ATRX-，EZH2-,H3K27M- |

Supplementary Table S1 Summary of Clinicopathological Characteristics of Glioma Patients

Supplementary Table S2 Clinical Data of Glioma Patients

| **Number** | **Admission Number** | **Age**  **(year)** | **Gender** | **PRS** | **Grade** | **1p19q**  **(+/-)** | **IDH**  **(+/-)** | **MGMT**  **(+/-)** | **KPS Score** | **Postoperative Chemotherapy** | **Postoperative Radiotherapy** | **Diagnosis date** | **Deathdate** | **Survival days** | **Survival state** |
| --- | --- | --- | --- | --- | --- | --- | --- | --- | --- | --- | --- | --- | --- | --- | --- |
| 1 | 443735 | 42 | M | P | Ⅳ | - | - | + | ＞80 | Yes | Yes | 2016/11/15 | NA | NA | dead |
| 2 | 434164 | 75 | F | P | Ⅳ | - | - | - | ＞80 | No | No | 2016/10/2 | 2016/10/25 | 23 | dead |
| 3 | 423888 | 26 | M | P | Ⅱ | - | - | + | ＞80 | Yes | Yes | 2016/11/8 | NA | NA | live |
| 4 | 433600 | 48 | F | P | Ⅳ | + | + | - | ＞80 | Yes | Yes | 2016/9/21 | NA | NA | live |
| 5 | 433535 | 45 | M | P | Ⅱ | + | + | - | ＞80 | Yes | Yes | 2016/9/27 | NA | NA | live |
| 6 | 432672 | 49 | M | P | Ⅱ | - | + | + | ＞80 | Yes | Yes | 2016/9/18 | NA | NA | live |
| 7 | 429712 | 27 | M | P | Ⅲ | - | + | - | ＞80 | No | No | 2016/8/31 | NA | NA | live |
| 8 | 430730 | 52 | F | P | Ⅱ | - | + | + | ＞80 | Yes | Yes | 2016/9/5 | NA | NA | live |
| 9 | 430590 | 47 | F | P | Ⅳ | - | - | - | ＞80 | No | No | 2016/9/4 | 2016/10/21 | 47 | dead |
| 10 | 422942 | 63 | M | P | Ⅱ | - | + | - | ＜80 | No | No | 2016/8/24 | 2019/10/21 | 1153 | dead |
| 11 | 254717 | 56 | M | S | Ⅲ | - | - | - | ＜80 | Yes | Yes | 2016/6/30 | 2016/11/5 | 128 | dead |
| 12 | 408689 | 57 | M | P | Ⅲ | - | - | - | ＜80 | Yes | Yes | 2018/5/15 | 2018/12/30 | 229 | dead |
| 13 | 187633 | 22 | M | P | Ⅱ | - | - | + | ＞80 | No | No | 2016/6/13 | NA | NA | live |
| 14 | 408981 | 60 | M | P | Ⅱ | + | + | - | ＞80 | No | No | 2016/5/16 | NA | NA | live |
| 15 | 404628 | 41 | M | P | Ⅲ | - | - | - | ＞80 | Yes | Yes | 2016/4/24 | 2018/5/2 | 738 | dead |
| 16 | 395393 | 53 | F | P | Ⅱ | - | - | + | ＞80 | No | No | 2016/3/9 | NA | NA | live |
| 17 | 397748 | 64 | M | P | Ⅲ | - | - | - | ＞80 | No | No | 2016/3/21 | 2016/6/3 | 74 | dead |
| 18 | 396796 | 51 | M | P | Ⅳ | - | - | + | ＞80 | No | No | 2016/3/15 | 2016/7/12 | 119 | dead |
| 19 | 359853 | 41 | M | P | Ⅳ | - | - | + | ＞80 | Yes | Yes | 2016/2/15 | 2019/3/12 | 1121 | dead |
| 20 | 388240 | 43 | M | P | Ⅳ | - | - | - | ＞80 | Yes | Yes | 2016/1/31 | 2018/3/18 | 777 | dead |
| 21 | 385325 | 55 | M | P | Ⅲ | - | - | - | ＞80 | No | No | 2016/1/31 | 2017/3/18 | 412 | dead |
| 22 | 381269 | 41 | F | P | Ⅱ | + | + | + | ＞80 | Yes | Yes | 2015/12/28 | NA | NA | live |
| 23 | 245756 | 30 | M | P | Ⅱ | - | + | + | ＞80 | Yes | Yes | 2015/4/17 | 2018/12/2 | 1325 | dead |
| 24 | 371419 | 51 | F | P | Ⅱ | + | + | + | ＞80 | Yes | Yes | 2015/11/5 | NA | NA | live |
| 25 | 373731 | 62 | M | P | Ⅳ | - | - | + | ＞80 | Yes | Yes | 2015/11/18 | 2016/9/6 | 293 | dead |
| 26 | 370778 | 30 | F | P | Ⅱ | - | - | + | ＞80 | Yes | Yes | 2015/11/2 | 2016/3/8 | 127 | dead |
| 27 | 370881 | 14 | F | P | Ⅱ | - | - | + | ＞80 | Yes | Yes | 2015/11/2 | 2020/10/8 | 1802 | dead |
| 28 | 361341 | 60 | M | P | Ⅲ | - | - | - | ＞80 | No | No | 2015/9/6 | 2015/10/10 | 34 | dead |
| 29 | 370131 | 41 | F | P | Ⅲ | - | - | - | ＞80 | No | No | 2015/10/10 | 2016/12/10 | 427 | dead |
| 30 | 365619 | 40 | M | P | Ⅲ | + | - | + | ＞80 | Yes | Yes | 2015/10/7 | 2018/5/25 | 961 | dead |
| 31 | 360268 | 54 | M | P | Ⅲ | - | - | + | ＞80 | No | No | 2015/9/29 | 2015/10/5 | 6 | dead |
| 32 | 361350 | 66 | F | P | Ⅳ | - | - | + | ＞80 | No | No | 2015/9/6 | 2016/9/10 | 370 | dead |
| 33 | 350201 | 64 | F | P | Ⅳ | - | - | + | ＞80 | Yes | Yes | 2015/7/8 | 2016/4/9 | 276 | dead |
| 34 | 347911 | 31 | M | P | Ⅳ | - | - | + | ＞80 | No | No | 2015/6/26 | 2016/4/15 | 294 | dead |
| 35 | 343637 | 54 | M | P | Ⅲ | - | - | + | ＞80 | Yes | Yes | 2015/6/3 | 2016/1/17 | 228 | dead |
| 36 | 342550 | 35 | F | P | Ⅳ | - | + | - | ＞80 | Yes | Yes | 2015/5/29 | 2017/2/5 | 618 | dead |
| 37 | 339234 | 46 | F | S | Ⅳ | - | + | - | ＞80 | Yes | Yes | 2015/5/12 | 2015/9/3 | 114 | dead |
| 38 | 341196 | 50 | F | P | Ⅱ | - | + | + | ＞80 | Yes | Yes | 2015/5/22 | 2018/9/10 | 1207 | dead |
| 39 | 329476 | 50 | F | P | Ⅱ | + | + | + | ＞80 | Yes | Yes | 2015/3/22 | NA | NA | live |
| 40 | 330537 | 40 | F | P | Ⅱ | - | + | + | ＞80 | Yes | Yes | 2015/3/27 | 2018/2/7 | 1048 | dead |
| 41 | 329996 | 42 | F | P | Ⅲ | + | - | - | ＞80 | Yes | Yes | 2015/3/24 | 2016/1/5 | 287 | dead |
| 42 | 326357 | 49 | M | P | Ⅳ | - | - | - | ＞80 | No | No | 2015/3/6 | 2019/5/15 | 1531 | dead |
| 43 | 319906 | 61 | M | P | Ⅲ | - | - | + | ＞80 | Yes | Yes | 2015/1/27 | 2015/6/12 | 136 | dead |
| 44 | 310849 | 66 | F | P | Ⅱ | + | + | + | ＞80 | Yes | Yes | 2014/12/12 | 2017/8/3 | 965 | dead |
| 45 | 307747 | 46 | F | P | Ⅲ | - | - | + | ＞80 | Yes | Yes | 2014/11/26 | 2019/5/23 | 1639 | dead |
| 46 | 304784 | 43 | M | P | Ⅳ | - | - | + | ＞80 | Yes | Yes | 2014/11/11 | 2015/4/2 | 142 | dead |
| 47 | 289471 | 37 | F | P | Ⅳ | - | - | + | ＞80 | No | No | 2014/8/16 | 2014/12/4 | 110 | dead |
| 48 | 290409 | 37 | M | P | Ⅲ | - | + | + | ＞80 | Yes | Yes | 2014/8/21 | 2017/5/23 | 1006 | dead |
| 49 | 286654 | 34 | M | P | Ⅱ | + | + | - | ＞80 | Yes | Yes | 2014/8/1 | 2019/10/20 | 1906 | dead |
| 50 | 254212 | 21 | M | P | Ⅱ | + | + | - | ＞80 | Yes | Yes | 2014/2/8 | 2018/3/14 | 1495 | dead |
| 51 | 257081 | 55 | F | P | Ⅲ | - | - | - | ＞80 | Yes | Yes | 2014/2/21 | 2015/10/21 | 607 | dead |
| 52 | 251809 | 63 | F | P | Ⅱ | + | + | - | ＞80 | Yes | Yes | 2014/2/8 | 2017/10/19 | 1349 | dead |
| Note: NA: not acquired(as of the date of statistics); F: Famale; M: Male; +means positive; -means negative. | | | | | | | | | | | | |  |  |  |
